# Isolating Anthropogenic Wetland Loss by Concurrently Tracking Inundation and Land Cover Disturbance across the Mid-Atlantic Region, U.S.

**DOI:** 10.3390/rs12091464

**Published:** 2020-05-05

**Authors:** Melanie K. Vanderhoof, Jay Christensen, Yen-Ju G. Beal, Ben DeVries, Megan W. Lang, Nora Hwang, Christine Mazzarella, John W. Jones

**Affiliations:** 1 Geosciences and Environmental Change Science Center, U.S. Geological Survey, Denver, CO 80225, USA; 2 Office of Research and Development, U.S. Environmental Protection Agency, Cincinnati, OH 45220, USA; 3 Department of Geography, Environment and Geomatics, University of Guelph, Guelph, ON N1G 2W1, Canada; 4 Department of Geographical Sciences, University of Maryland, College Park, MD 20740, USA; 5 National Wetlands Inventory Program, U.S. Fish and Wildlife Service, Falls Church, VA 22041, USA; 6 Region 5, Water Division, Wetlands Section, U.S. Environmental Protection Agency, Chicago, IL 60604, USA; 7 Region 3, Water Division, Wetlands Branch, U.S. Environmental Protection Agency, Philadelphia, PA 19103, USA; 8 Hydrologic Remote Sensing Branch, U.S. Geological Survey, Leetown, WV 25430, USA

**Keywords:** Chesapeake Bay, wetland fill, harmonic regression, Landsat, permit, surface water

## Abstract

Global trends in wetland degradation and loss have created an urgency to monitor wetland extent, as well as track the distribution and causes of wetland loss. Satellite imagery can be used to monitor wetlands over time, but few efforts have attempted to distinguish anthropogenic wetland loss from climate-driven variability in wetland extent. We present an approach to concurrently track land cover disturbance and inundation extent across the Mid-Atlantic region, United States, using the Landsat archive in Google Earth Engine. Disturbance was identified as a change in greenness, using a harmonic linear regression approach, or as a change in growing season brightness. Inundation extent was mapped using a modified version of the U.S. Geological Survey’s Dynamic Surface Water Extent (DSWE) algorithm. Annual (2015–2018) disturbance averaged 0.32% (1095 km^2^ year^−1^) of the study area per year and was most common in forested areas. While inundation extent showed substantial interannual variability, the co-occurrence of disturbance and declines in inundation extent represented a minority of both change types, totaling 109 km^2^ over the four-year period, and 186 km^2^, using the National Wetland Inventory dataset in place of the Landsat-derived inundation extent. When the annual products were evaluated with permitted wetland and stream fill points, 95% of the fill points were detected, with most found by the disturbance product (89%) and fewer found by the inundation decline product (25%). The results suggest that mapping inundation alone is unlikely to be adequate to find and track anthropogenic wetland loss. Alternatively, remotely tracking both disturbance and inundation can potentially focus efforts to protect, manage, and restore wetlands.

## 1. Introduction

Across the globe, wetlands provide a myriad of ecosystem services including flood abatement, erosion control, hydrologic regulation, carbon sequestration, and water quality improvement [1–3]. Wetland ecosystems are threatened, however, by expansion of agriculture and urban development, invasive species, pollution, and sea level rise, all of which can result in wetland degradation or loss [4,5]. Globally, as much as 87% of wetland area has been lost since 1700 CE [6], and 30% since 1970 [7]. The Ramsar Convention on Wetlands of International Importance was established in 1971 in recognition of the value of and threats to wetlands. It included policy commitments to maintain and restore wetlands [6]. However, even in countries such as the United States, which has a national policy of no net loss of wetlands, mitigating losses through wetland creation and restoration can fall short, resulting in net wetland degradation and declines in wetland area [8,9]. These trends make it essential to monitor wetland extent over time, as well as enhance our understanding of the causes and spatial distribution of wetland loss and gain.

Satellite imagery can provide a spatially explicit and cost-effective means to monitor changes in wetland extent and patterns in wetland loss over time [10,11]. Landsat is commonly used to monitor wetlands in part because of its multi-decadal temporal archive and substantial swath (185 km). The National Oceanic and Atmospheric Administration (NOAA) Coastal Change Analysis Program (C-CAP), for example, uses Landsat imagery to map changes in land cover for the coastal portions of the United States every five years (1996–2016) [12]. To provide a more temporally detailed understanding of seasonal and interannual variability in the surface water extent of wetlands, Landsat-based surface-water products at national and global extents are also rapidly becoming available. These efforts include the Global Surface Water (GSW) Landsat product [13] and the U.S. Geological Survey’s (USGS) Dynamic Surface Water Extent (DSWE) product [14,15] that use the Landsat archive (available every 8–16 days) to map surface water extent on a global and contiguous United States (CONUS) scale, respectively. The more frequent monitoring is critical to characterize variability of inundation in wetlands in response to seasonal and interannual variability in weather [16], as well as variability in coastal wetlands in response to the tidal cycle [17].

Landsat multispectral data have also been used to distinguish wetland classes, such as emergent wetlands, forested wetlands, and ponds [18,19], as well as monitor the degradation or recovery of wetland vegetation [20,21]. Detecting a gain or loss of wetland extent requires distinguishing a change in class from natural variability in pixel condition. The likelihood that a pixel has changed its class can be quantified as its departure from a measure of central tendency for all pixels in that class. A z-score statistic, for example, that uses the mean and standard deviation of a population, is one way to achieve this [22,23]. The challenge in applying z-scores over a large area, however, is that the population must be normally distributed, which may not be true as more images and a greater area is included in the analysis [24]. Alternatively, measures such as median and median absolute deviation do not rely on the assumption of a normally distributed population and are less sensitive to outliers [24]. Improvements in using Landsat time series analysis to detect change have also been specific to different land cover types. For instance, in forestry studies the focus has been on using harmonic algorithms that model interannual and seasonal variability in spectral characteristics to detect both abrupt changes in forest cover [25–28], as well as gradual changes in forest condition [29,30]. Efforts in wetland ecosystems, in contrast, have been more focused on overcoming Landsat’s moderate spatial resolution that limits its ability to monitor smaller wetlands (<1 ha) [10]. This challenge has encouraged Landsat sub-pixel analysis techniques, such as spectral mixture analysis (SMA) [15,31,32] and Tasseled Cap indices [33,34], as well as other partial pixel algorithms, such as regression trees [35], or automated methods to estimate sub-pixel water fraction [36–38]. Even sub-pixel Landsat approaches, however, typically require wetlands to be >0.2 ha to be reliably detected [32,37].

To monitor smaller wetlands (<1 ha), data sources with increased spatial resolution have been used (e.g., Sentinel-2, CubeSats) [39,40]. However, these datasets lack the multi-decadal data records provided by Landsat. Additionally, the cost of commercial imagery (e.g., DigitalGlobe, Planet) may limit its role in the operational monitoring of wetlands. Free sources of aerial imagery, such as the National Agricultural Imaging Program (NAIP), have been shown to be an effective means to monitor interannual variability in wetlands over time [41]. However, the applicability of these data to regions dominated by forested or vegetated wetlands may be limited. Instead, synthetic aperture radar (SAR) imagery may be more helpful to “see” water through canopy cover [42–44].

The persistent threats to wetlands necessitate efforts to monitor changes in wetland extent at the regional to national scale. Past efforts to monitor changes to coastal and inland wetlands across the U.S. Mid-Atlantic region have tended to be mostly localized, focusing on one or a few watersheds. Studies have explored approaches to map forested wetlands using active sensors, such as C-band SAR [42,45], L-band SAR [46], LiDAR [47], and Landsat trained with LiDAR [48,49]. Efforts have also focused on monitoring tidal wetlands in the Chesapeake and Delaware Bays using aerial imagery [6,50] and Landsat imagery [21,51]. Regional efforts have used data from the U.S. Fish and Wildlife Service’s Status and Trends Study (S&T) to predict change in wetland area between the 1950s and 1990s along the U.S. Atlantic Coast [52], and comparisons between the National Wetland Inventory (NWI) and Landsat circa 2000 [24]. In general, although efforts to monitor wetland change over time are somewhat common [14,32], relatively few studies have explicitly attempted to attribute the cause of the change, for instance differentiating between wetland loss caused by a drought event versus human activities [9,31,34,53]. Identifying both the spatial distribution of modifications to wetland extent as well as the cause of wetland change are critical for modeling, management, and conservation efforts. The objectives of this study were to determine (1) if wetland loss, defined as a transition from wetland to upland, can be reliably detected using changes in Landsat inundation extent; (2) if mapping disturbance extent across all land cover types can enable the identification and attribution of wetland loss; and (3) the spatial distribution of disturbance and changes to inundation extent across the Mid-Atlantic region.

## 2. Materials and Methods

### 2.1. Study Area

The study was limited to the U.S. Environmental Protection Agency (EPA) Region 3, which extends across the Mid-Atlantic region of the United States, including Pennsylvania, Delaware, Maryland, Washington, D.C., Virginia, and West Virginia (study area center: 39.403 N, −77.974 W). The climate across these states is temperate with annual precipitation averaging 1122 mm, and annual temperature maximum and minimum averaging 17 °C and 5 °C, respectively [54]. Across the region, deciduous forest is the dominant cover type (51%), with pasture/hay (13%), developed land (open space, low, medium, high) (11%), and cultivated crops (7%) also common [55]. Developed land dominates around major cities within the study area, including Washington, D.C., Baltimore, Virginia Beach, Richmond, Philadelphia, and Pittsburgh. Wetlands, as defined by the NWI dataset, average 5.1 ha km^−2^ across the region and wetland area is dominated by freshwater forested/shrub wetlands (46%) and riverine wetlands (26%), with lakes (9%), estuarine and marine wetlands (8%) and freshwater emergent wetlands (6%) also common [56].

### 2.2. Inundation Extent

The Landsat archive was used to map annual inundation extent across the region. Although the pixel size of Landsat Enhanced Thematic Mapper plus (ETM+) and Operational Land Imager (OLI) (900 m^2^) makes monitoring inundation extent across narrow rivers, streams, and smaller (<1 ha) wetlands challenging, the source of imagery was selected to take advantage of regular (every 8 days), wall-to-wall data collection. Mapping surface-water or inundation extent cannot be considered equivalent to mapping wetlands, but areas that are regularly detected as inundated are very likely to meet the hydrologic definition of a wetland (i.e., inundated or saturated in the root zone for at least two weeks within the growing season) [57]. In limiting the detection algorithm to inundation, however, areas that show near-surface saturated soils were likely omitted, although these areas may also meet wetland definitions. A further challenge was that much of the Mid-Atlantic region and the wetlands across the region are forested, making it more difficult to detect inundation under leaf cover with multispectral imagery. Prior efforts have shown, however, that inundation extent can still be monitored using Landsat imagery if the acquisition dates include leaf-off conditions [45,49].

#### 2.2.1. Single Image Inundation Classification

Inundation extent across the region was mapped with Landsat ETM+ and OLI imagery using a modified version of the image-based components of the Dynamic Surface Water Extent (DSWE) model [15] implemented in Google Earth Engine. Landsat imagery was used from Landsat paths 14 to 19 and Landsat rows 31 to 35 (a total of 30 Landsat path/rows). All Landsat images collected between January 1 and May 31 from 2013 through 2018 for these path/rows were processed for a total of 2122 Landsat images (Table 1). The seasonal restriction of images helped maximize images used during the seasonal hydrological peak [58], while limiting the number of images contributing errors during non-peak hydrological conditions. Landsat 5 and 7 images were converted to surface reflectance using the Landsat Ecosystem Disturbance Adaptive Processing System (LEDAPS) algorithm [59], while Landsat 8 images were converted to surface reflectance using the Landsat Surface Reflectance Code (LaSRC) algorithm [60]. Values identified as cloud or cloud shadow were masked using cFMask [61].

The DSWE model detects inundation using a single-scene approach through a series of static thresholds applied to combinations of indices and band values to ultimately label pixels into one of four inundation classes: (1) inundation—high confidence, (2) inundation—moderate confidence, (3) partial surface water/wetlands—conservative, and (4) partial surface water/wetland—aggressive/low confidence. An advantage of the DSWE approach is that it is essentially an unsupervised classifier, meaning it does not require scene-specific training data and therefore can be easily applied across space and time. The thresholds in the model were based on spectral unmixing and evaluation of results over a range of water landscapes across North America [14,15]. Individual bands used in the classification include blue, near infrared (NIR), shortwave infrared one (SWIR1), and shortwave infrared two (SWIR2). Indices used include modified Normalized Difference Wetness Index (mNDWI = (Green − SWIR1)/(Green + SWIR1) [62], NDVI [63], the Multi-band Spectral Relationship Visible (MBSRV = (Green + Red) − (NIR + SWIR1) [64], and the Automated Water Extraction Shadow (AWEsh = Blue + 1.5*Green – 1.5*(NIR + SWIR1) − 0.25*SWIR2) [65]. The bands and indices are organized into five tests applied to each pixel (Table 2), and the inundation confidence class is then based on the combination of tests for which water detection is “positive” [15]. In applying the image-based component of the DSWE model to Landsat ETM+ images several minor modifications were made. To reduce confusion between upland forests and forested wetlands an additional NDVI requirement of <4000 was added to Test 5 and the NDVI threshold was changed to be more conservative (i.e., <0.6 rather than <0.7) in Test 4 (Table 2).

Version 1 of DSWE was trained on Landsat TM and ETM+ only. When the DSWE algorithm was applied to Landsat 8 in the study area, a decline in forested wetland extent was observed, as well as an increase in commission error in areas of low to medium density development. To enhance the detection of forested wetlands an additional Test (i.e., Test 6) was added to the DSWE model where wetlands were found if Green<490, NIR<2500, and NDVI <5500 (Table 2). These threshold values were selected using a classification tree in R (rpart package) using points randomly selected across the study area (deciduous and coniferous forest (n = 588), agriculture and grassland (n = 463), urban (n = 405), and forested wetlands (n = 368)). Band and index values were extracted from six Landsat 8 images (p14r33: November 16, 2016, November 10, 2017, April 28, 2018, July 1, 2018; p15r34: April 16, 2017, November 29, 2018). To reduce confusion with suburban and urban development, the 3 Band Index was calculated where BU3 = red + SWIR1 − NIR and pixels with BU3 ≥ 1600 were classified as urban [66]. This threshold requirement (BU3 < 1600) was added to Landsat 8 Test 5 and 6.

For each image considered, a pixel was classified as low to moderate confidence inundated if it passed at least two of the Tests, except for Test 5 and Test 6, where if either Test 5 or 6 was satisfied, it was classified as inundated. High confidence inundation was defined as pixels that passed four or more of the tests. Lastly, as modification to the terrain-based components of the DSWE model [67], the percent slope was calculated using a USGS digital elevation model (DEM, 30 m) derived from the Shuttle Radar Topography Mission (SRTM) [68] and inundation was masked from areas with a slope ≥7%. Although this mask may have reduced the detection of springs that can occur on slopes, it greatly reduced errors from topographic shadowing that were particularly prevalent in the Appalachian Mountains that dominate West Virginia, western Virginia, and central Pennsylvania.

#### 2.2.2. Annual Inundation Extent

**Figure 1.**
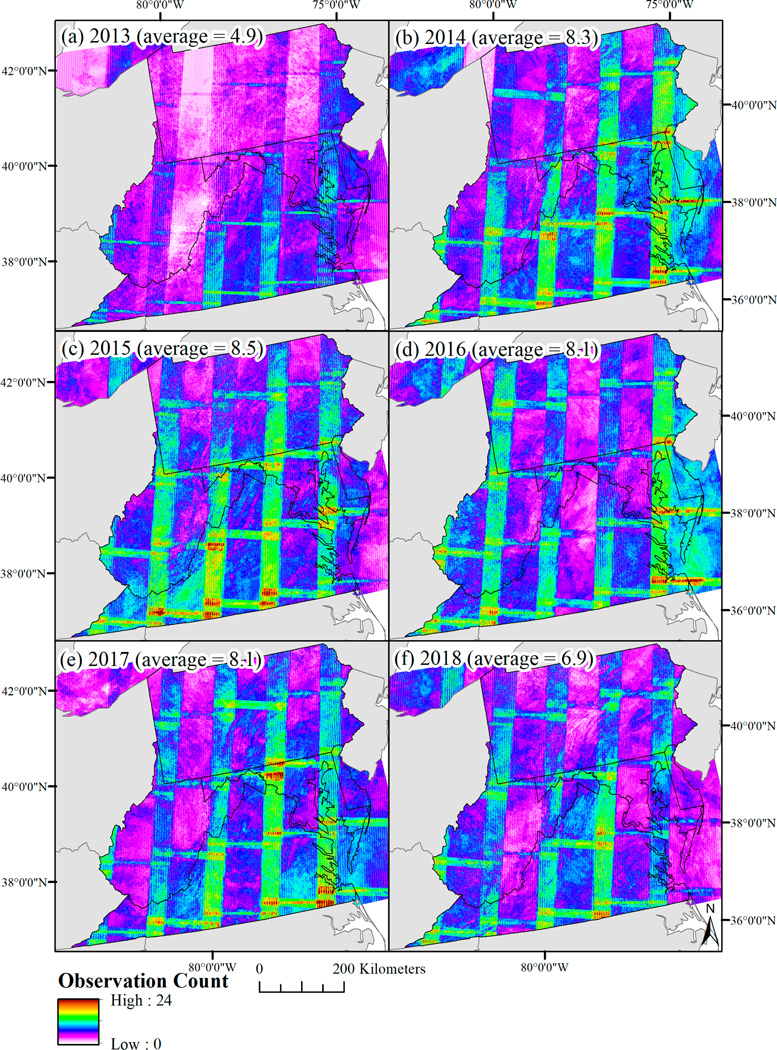
The per-pixel and per-year observation count for January 1 through May 31 integrating Landsat Enhanced Thematic Mapper plus and Landsat Operational Land Imager, after excluding cloud and cloud shadow observations.

The modified DSWE model calculated inundation extent (and confidence class) per image. The per-image classification was then applied to all Landsat images across the study area and time period of interest (2013–2018). On an annual time-step, a raster was produced representing the number of inundation observations for each inundation confidence class per year. The primary challenge in reclassifying the annual inundation count to an annual binary inundation/non-inundation extent was to limit image-based error contributions while retaining inundation extent for small, ephemeral, forested wetlands common across the Delmarva Peninsula. For example, the number of cloud-free observations across the study area for a given year was highly uneven, and commission error tended to be higher in the overlapping areas between Landsat path/row images where the number of cloud-free observations tended to be double or triple the number of cloud-free observations found elsewhere (Figure 1).

**Figure 2.**
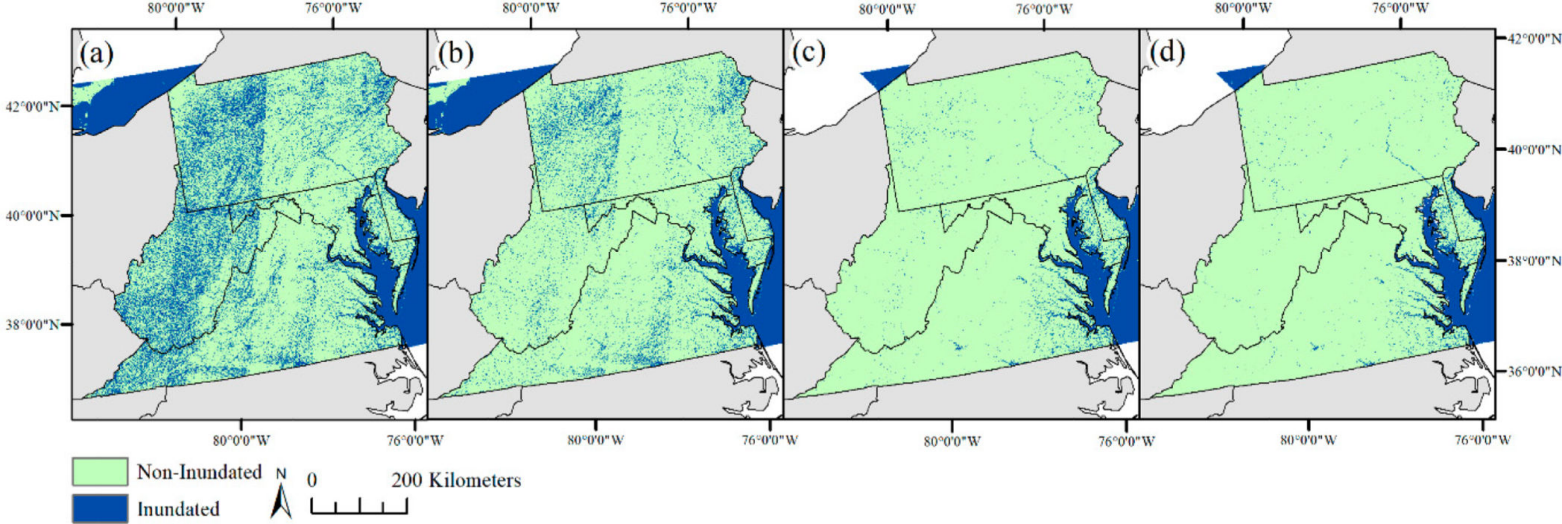
Inundation extent in 2016 as defined by (**a**) two or more observations of low, moderate, or high confidence inundation, (**b**) inundation extent in *a* with a slopes greater than 7% masked out, (**c**) inundation extent in *b* with additional observations required of low or moderate confidence inundation outside of the coastal plain ecoregions, and (**d**) extent in *c* with inundation polygons required to also intersect a National Wetlands Inventory polygon.

The accuracy of the annual products was maximized by considering: (1) inundation confidence level, (2) ecoregion, (3) the number of times a pixel was classified as inundation per year, and (4) the number of cloud-free observations. Across the entire study area, a pixel was classified as inundated when there were (1) at least two high confidence inundation observations in a given year, (2) at least six observations of low to moderate confidence inundation in a given year when the total observation count was <14, or (3) at least eight observations of low to moderate confidence inundation in a given year when the total observation count was >14. In the lowlands, this was expanded to classify pixels as inundated when there were at least two observations of either high confidence inundation or low to moderate confidence water each year. The lowlands were defined using a modified version of the U.S. Environmental Protection Agency Level III Ecoregion definitions [69]. Lowland Ecoregion extent included the Middle Atlantic Coastal Plain, Southeastern Plains, Eastern Great Lakes Lowlands, Lake Erie, Chesapeake Bay, and the Atlantic Ocean [69]. Upland Ecoregion extent included the Erie Drift Plain, the Appalachians, Blue Ridge, Piedmont, Northern Piedmont, and Western Allegheny Plateau [69]. Lastly, only inundated polygons that overlapped an NWI wetland [56] were retained to further eliminate erroneously mapped inundation. An example showing the reduction of inundation extent error through each processing step is shown in Figure 2.

**Figure 3.**
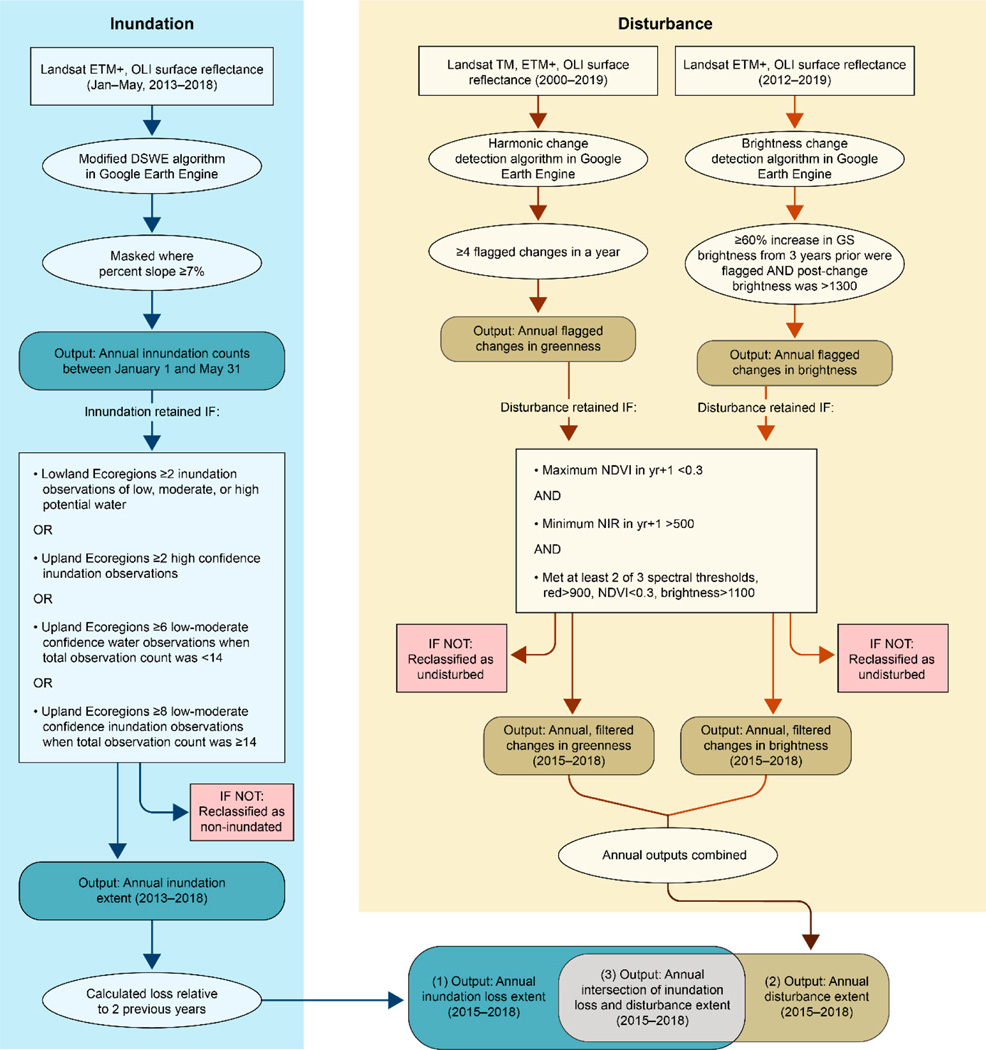
A summary of the steps taken to produce the annual inundation loss, annual disturbance extent and annual intersection of these two products. TM: Thematic Mapper, ETM+: Enhanced TM, OLI: Operational Land Imager, DSWE: Dynamic Surface Water Extent, GS: Growing Season, NDVI: Normalized Difference Vegetation Index, NIR: Near Infrared.

Inundation loss (2015–2018) in this study was defined as a pixel that was mapped as inundated in either of the two previous years but was not mapped as inundated in the present year. Change from two years was used to increase the probability of identifying anthropogenic inundation loss in a year following a dry year. The area identified as “loss” therefore includes: (1) natural variability in inundation extent, (2) underestimated inundation extent in the current year due to cloud cover during peak wetness conditions, (3) erroneously identified inundation mapped in prior years that is correctly mapped in the current year, and (4) permanent or semi-permanent water loss due to anthropogenic activities. An overview of the processing steps is shown in Figure 3.

### 2.3. Disturbed Extent

Disturbance extent was mapped across all land cover types in Google Earth Engine using a combination of two approaches, first a harmonic regression approach, and second by tracking changes in pixel brightness. Annual, binary image classifications were created representing (1) disturbed extent (e.g., conversion of water or vegetation to bare soil, removal of overstory vegetation, water fill), and (2) non-disturbed extent (e.g., grassland, agriculture, forest, wetland, open water, or consistent bare soil). The harmonic linear regression used all per-pixel observations of the Normalized Difference Vegetation Index (NDVI) from Landsat TM, Landsat ETM+, and Landsat OLI surface reflectance from January 2000 through December 2018 (Table 1). Values identified as cloud or cloud shadow were masked using the cFMask [61]. The NDVI observations were used to train a per-pixel harmonic linear regression that used the seasonal variability in NDVI (one harmonic cycle per year) to predict the NDVI values over the same period (2000–2018). An observation was flagged when the NDVI value deviated substantially from the expected NDVI variability, defined as three times a pixel’s root mean square error (RMSE). The RMSE was calculated using the difference between the observed and expected NDVI over the time series. Further details of the algorithm are explained in Zhu and Woodcock [25]. Deviations from the Zhu and Woodcock [25] approach included: (1) flagging NDVI observations that were >70% of the 3 × RMSE instead of >100% of 3 × RMSE, and (2) requiring four flagged observations, instead of three, to indicate a change in a pixel. Multiple flagged observations were required to limit the influence of erroneous Landsat images flagging change falsely. To further limit the inclusion of erroneous flags only flagged anomalous observations acquired between March and November were retained.

**Figure 4.**
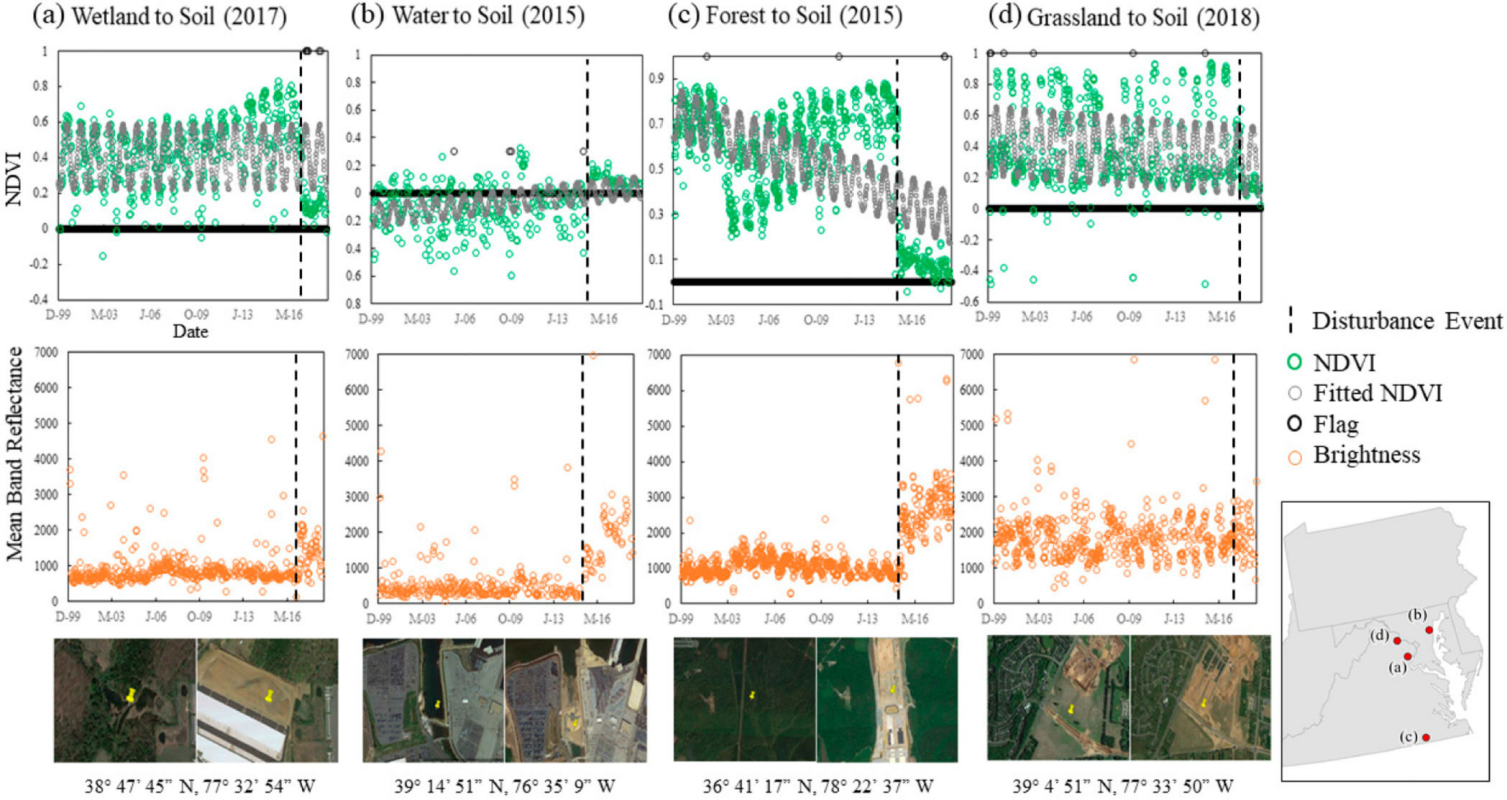
Variability was observed in the performance of the harmonic linear regression (top) and change in brightness (middle) to document transitions to bare soil. Examples shown include (**a**) a wetland fill found using the harmonic linear regression, (**b**) a water fill and (**c**) forest to soil transition, found using the increase in brightness, and (**d**) a transition from grassland to bare soil, missed by both approaches. Images are from Google Earth Pro. NDVI: Normalized Difference Vegetation Index.

Although NDVI seemed to outperform other indices in preliminary investigations, in multiple scenarios a conversion to bare soil was missed by the harmonic linear regression approach. The predicted NDVI values in some cases underestimated the pre-change NDVI observations so that despite a substantial change in NDVI values, the post-change NDVI values were found to be within the allowable RMSE variability. When pre-change NDVI values were negative or highly variable (e.g., open water), the harmonic regression approach also frequently did not identify change as expected. Examples showing change in NDVI flagged as expected and as unexpected are shown in Figure 4. To compensate for scenarios in which the harmonic linear regression did not identify conversion to bare soil, change in pixel brightness was tracked in growing season (May–September), where brightness was defined as the mean band reflectance across six spectral bands (blue, green, red, NIR, SWIR1, and SWIR2). Per-pixel surface reflectance observations from Landsat TM, Landsat ETM+, and Landsat OLI between June 2012 and September 2018 were used. Values identified as cloud or cloud shadow were masked using the cFMask [61]. An increase in growing season (June–September) pixel brightness of >60% relative to the average growing season brightness from the previous three years (e.g., 2015 brightness relative to 2012–2014 brightness) concurrent with a post-change brightness of ≥1300 was flagged as a change. For both analyses, all images were internally buffered by −500 m to eliminate erroneous values and noise that tended to occur near the edges of the Landsat images.

#### Classifying Change as Disturbance

A change to a pixel’s NDVI or brightness could, of course, indicate several state transitions including, but not limited to, deforestation, afforestation, or an increase or decrease in inundation extent. Change in the pixel condition, identified by either the harmonic linear regression or the brightness analysis, that did not represent a conversion to disturbance, was limited by: (1) establishing a spectral window defining disturbed pixels, (2) masking change pixels where the maximum NDVI in the year following the change was >0.3 to limit highly temporary change or shifts in the timing of crop patterns, and (3) masking change pixels where the minimum NIR value in the year following the change was <500 to limit the inclusion of turbid water or natural variability in open water extent. The spectral window required pixels flagged as changed to only be retained if they met at least two of three spectral thresholds at the time of the change: (1) red band reflectance >900, (2) NDVI <0.3, and (3) average band reflectance (or brightness) of >1100. All spectral thresholds were derived using class separability and natural breaks in the spectral values of training points (n = 10,971) selected from manually delineated polygons representing disturbed, forest, grassland, agriculture, open water, wetlands, and impervious surfaces. The polygons were delineated from Landsat images representing spring (March–May), summer (June–August), and autumn (September–November) spectral conditions across five Landsat path/rows (p15r31, p15r33, p16r34, p17r32, p18r34) (Table A1). After applying the post-change spectral conditions, the remaining pixels classified as disturbed were compiled over time to produce an annual raster (2015–2018) of change. A summary of the processing steps is shown in Figure 3.

### 2.4. Validation

Independent validation efforts were used to validate: (1) inundation extent, (2) conversion to bare soil, and (3) waterbody fill. Sampling was stratified by: (1) inundation class and (2) disturbance class. Because the rare cover type (i.e., inundation, disturbance) was the cover type of interest, sample points were equally allocated between the two strata.

#### 2.4.1. Validation of Inundation Extent

**Figure 5.**
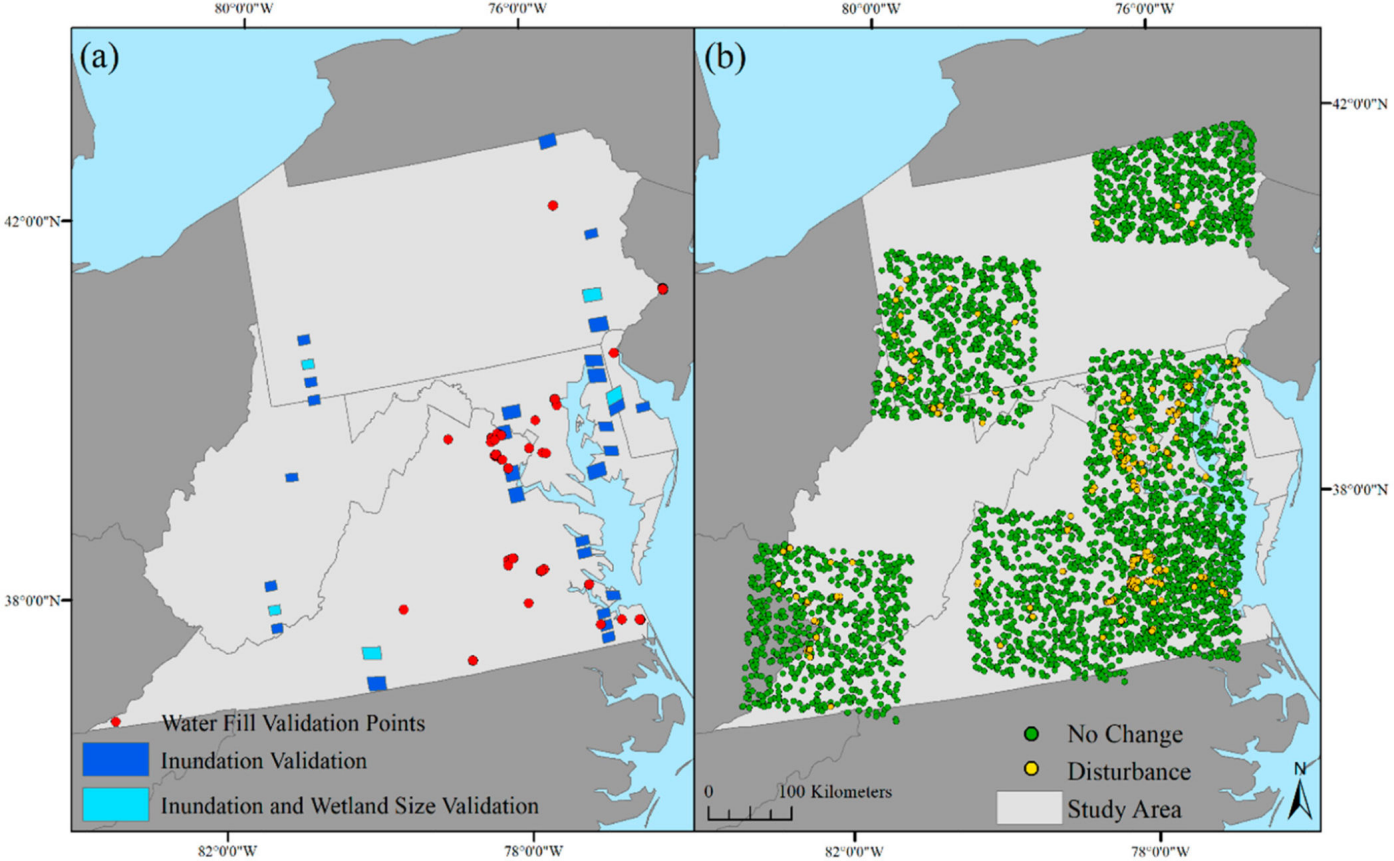
The distribution of (**a**) WorldView-2, 3 image extent used to validate inundation extent, minimum wetland size, and water fill validation points, and (**b**) disturbance extent validation points.

To validate the Landsat inundation extent, 8-band WorldView-2 (2 m resolution) and WorldView-3 (1.4 m resolution) images (n = 32) were obtained from DigitalGlobe via the NextView License (Westminster, CO) (Table A2, Figure 5a). The availability of WorldView-2 and WorldView-3 imagery was uneven across the study area. Images were selected to: (1) minimize the temporal gap between a cloud-free Landsat ETM+ and Landsat OLI image and the high-resolution image, (2) represent diverse vegetation types, and (3) approximate the spatial distribution of inundation across the Mid-Atlantic states, which occurs disproportionately in the eastern portion of the study area. The absolute average date gaps between the high-resolution image acquisition date and the Landsat ETM+ and OLI collection dates was 9.2 days (ranged from 0 to 38 days apart) and 11.1 days (ranged from 0 to 36 days apart), respectively. Using ENVI (Harris Geospatial Solutions, Inc., Broomfield, CO), the WorldView-2 and 3 images were converted to radiance and atmospheric conditions were taken into account using dark object subtraction. The WorldView water index (WV-WI = (coastal – NIR2)/(coastal + NIR2)) [70] and Red-Edge index (RE68 = (red edge – NIR2)/(red edge + NIR2)) were calculated. These indices showed the maximum class separability between open water and non-open water and wetlands and non-wetlands, respectively. Using these two indices as inputs, a maximum likelihood classification was calculated. Two to four training polygons were selected per image to represent the major cover classes across the image. A Frost filter was applied using a kernel size of 3 and an aggregated minimum size of 10 pixels. The classified raster was then reclassified to represent inundation and non-inundation. For each classified image, random points were generated across the inundation (n = 250) and non-inundation (n = 250) categories (total points = 16,000). The points were then visually checked against the raw high-resolution image to ensure that the correct classification was assigned to each point. If the point was incorrectly assigned, it was deleted. Landsat inundation extent was not shared with interpreters to avoid bias. In addition to validating inundation extent, the minimum size of wetlands reliably detected, or the minimum mapping unit, was also evaluated. A subset of five WorldView validation images was selected (Figure 5A). The WorldView image classifications were converted to polygon shapefiles where an overlap between the per-date Landsat (ETM+ and OLI) inundation extent and the WorldView wetland polygons was considered detection. Wetlands where the corresponding Landsat image was cloudy were excluded from consideration.

#### 2.4.2. Validation of Disturbance Extent

Creation of a reference dataset of conversion to bare soil imagery with a regular collection interval was needed so that both pre- and post-disturbance conditions were observable. WorldView imagery is only collected on demand and therefore would not necessarily provide pre-disturbance conditions. Six Landsat path/rows were selected across the study area (p15r31, p15r33, p15r34, p16r34, p17r32, p18r34), and one to three cloud-free Landsat OLI images per year were acquired within each of these Landsat path/rows (Table A3). Note that the approach of deriving validation data from Landsat to validate a Landsat product has been used by a number of studies when steps have been taken to increase the accuracy, specifically, manually evaluating the imagery [71–74]. Across each path/row extent 150 points per year (2015–2018) were randomly generated to represent “no-change” points. The points were visually checked with the prior year images to verify that no conversion to bare soil had occurred. Ancillary datasets were used to assist in finding areas that had been converted to bare soil, including the U.S. Army Corps of Engineers, Jurisdictional Determinations and Permit Decisions database, and the active mines and mineral plants point shapefile monitored by the National Minerals Information Center of the USGS. Polygons were manually delineated that showed new disturbance that was not visible in previous year images. From these polygons, points were randomly generated representing conversion to disturbance. As the amount of land cover change was uneven across the study area and years, the number of random points representing disturbance also varied (total disturbed points = 2711) (Figure 5b). A minimum distance of 30 m was required between all points, while the distance between points averaged 2.1 km. No co-occurrence of the training points was used to establish spectral filters and the disturbed validation points. Timing of the change relative to the image acquisition dates could have introduced error into the reference dataset (e.g., point classified as no change based on a July Landsat image when a change occurred in October). To compensate for this uncertainty, a −1 to +1-year buffer was applied when matching the validation point class to the mapped output (e.g., a point identified as new bare soil in 2016 could map the point as new bare soil in 2015–2017).

#### 2.4.3. Validation of Water Fill

Points where water resources had been filled were identified using the U.S. Army Corps of Engineers (USACE) ORM Jurisdictional Determinations and Permit Decisions database. This database identifies a project location and the date a permit was issued. Permits issued in 2015–2018 were downloaded for five USACE districts including (1) Huntington District, (2) Pittsburgh District, (3) Norfolk District, (4) Baltimore District, and (5) Philadelphia District. Project locations occurring within the EPA Region 3 states were exported to Google Earth Pro. Historical aerial imagery time series available within Google Earth Pro was used to manually delineate the extent of conversions from inundation (e.g., loss of streams, rivers, ponds, or wetlands). Project locations were excluded from further consideration where: (1) construction or disturbance was not visible (i.e., construction had not yet occurred or was limited to dredging of open water or installation of a dock); (2) the conversion was from non-water or wetland to open water; or (3) a disturbance was visible, but no aquatic features were visible. Random points (n = 263) with a minimum distance between the points of 30 m were generated to represent loss of ponds or open water (n = 68), streams and rivers (n = 65), and wetlands (n = 130) (Figure 5a).

### 2.5. Ancillary Datasets

Climate conditions were quantified using the monthly Palmer Hydrological Drought Index (PHDI), calculated from precipitation and temperature station data and interpolated at 5 km [75]. To quantify climate conditions at the seasonal peak in inundation extent, March and April PHDI values were averaged across the region. In addition to examining Landsat-based inundation extent, the distribution of aquatic resources as defined by the NWI dataset, version 2, Surface Waters and Wetlands Inventory, was also considered [56]. This dataset was derived from fine spatial resolution satellite or aerial imagery and designed to represent wetland extent under “average” hydrological conditions [56]. The advantage of including this dataset is that potential impacts to wetlands and streams smaller or narrower than reliably detected with Landsat can be examined and mapped. The co-occurrence between inundation loss or NWI data and the disturbance was defined as the annual intersection of the two inputs. To account for mixed disturbance pixels, the disturbance extent was internally buffered by 30 m and recalculated as the intersection between the disturbance extent and NWI dataset. To quantify the distribution of change by watershed position, the Strahler Order/Strahler Calculator (SOSC) was linked to the National Hydrography Dataset Plus catchment polygon dataset. Headwater extent was defined as the contributing areas of all first-order streams across the study area [76].

## 3. Results

### 3.1. Accuracy of Outputs

In comparing Landsat ETM+ and OLI inundation extent relative to the inundation extent derived from processed WorldView-2, 3 imagery, errors of omission for inundation (17.5% and 18.7%, respectively) were larger than errors of commission for inundation (0.9% and 4.3%, respectively) (Table 3). When the Landsat ETM+ and OLI inundation extent was merged for each high-resolution image and date, errors of omission for inundation decreased to 12.6%, suggesting that using both sources of imagery together, as is done in producing the annual inundation extent, produces a more complete inundation extent (Table 3). In determining the minimum wetland size reliably mapped, a total of 1176 wetlands >900 m^2^ in size were evaluated across five WorldView images (median size = 0.2 ha). The per-image Landsat (ETM+ and OLI) inundation extent mapped 61% of wetlands between 0.4 and 1.0 ha in size, and 84% of wetlands between 1.0 and 1.5 ha in size.

In validating the annual disturbance extent, the harmonic and brightness approaches alone showed relatively high rates of omission error for disturbance (27.0% and 56.1%, respectively), but the methods were highly complementary so that defining disturbance as detected by either the harmonic or brightness approach resulted in an error of omission of 15.5%, while maintaining a low commission error (1.9%) (Table 4). Of the 263 USACE permitted water fill points, 95.1% of the points were detected by either or both disturbance and inundation loss products. By output type, 70.7% of the points were detected using the disturbance outputs alone, 6.5% detected using inundation loss outputs alone, and 17.9% detected by both the disturbance and inundation loss outputs. By water feature type, 66 of 68 pond loss points, 62 of 65 stream loss points, and 122 of 130 wetland loss points were detected.

### 3.2. Change Analysis

**Figure 6.**
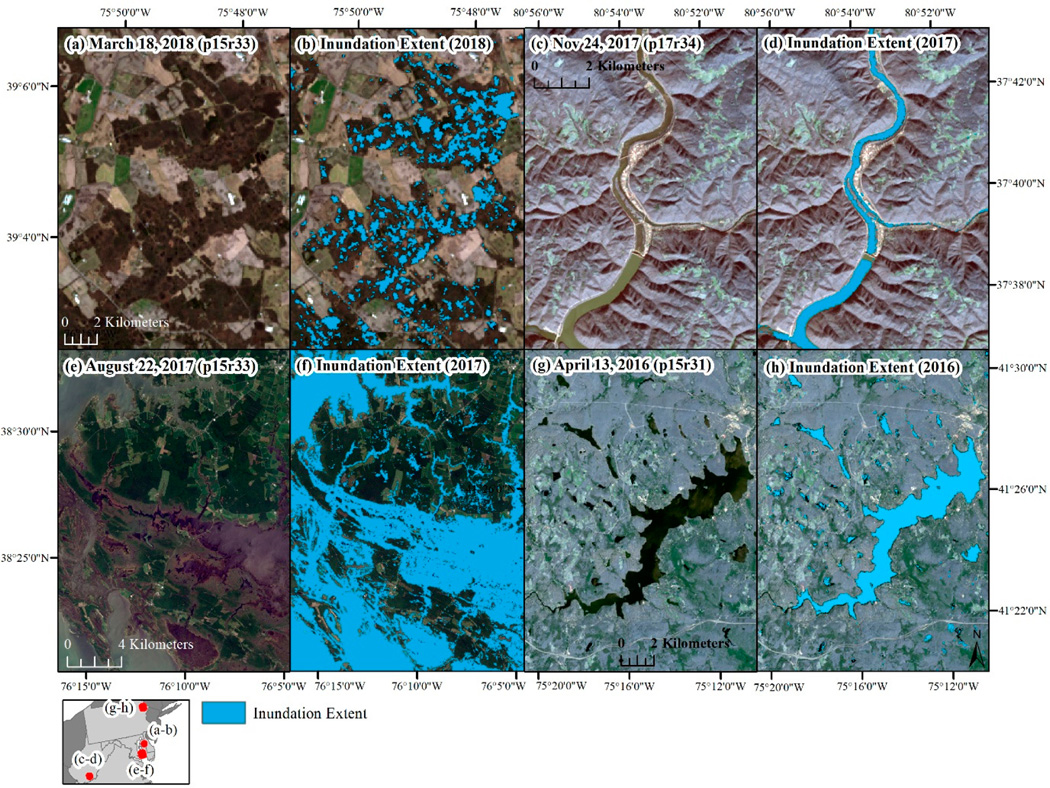
Annual inundation extent examples across (**a**,**b**) forested wetlands, (**c**,**d**) river extent, (**e**,**f**) tidal emergent wetlands, (**g**,**h**) lake and emergent wetlands.

**Figure 7.**
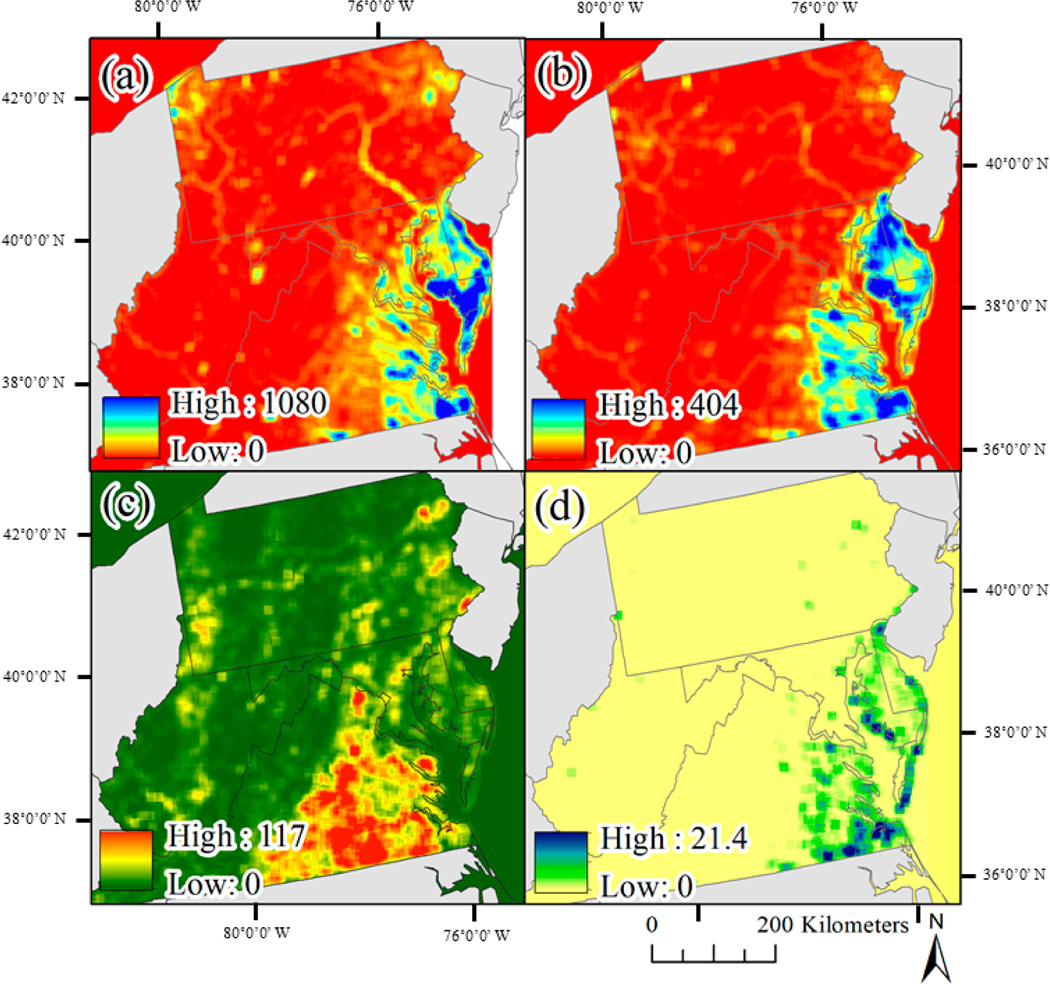
(**a**) The density of National Wetland Inventory (NWI) wetlands, the density of (**b**) water loss, (**c**) disturbance, and (**d**) the intersection of disturbance and water loss. For a-d density was calculated from 5 × 5 window of cells using a 3 km resolution, for b-d density was based on the summed 2015–2018 annual outputs.

Across the six years in which inundation was mapped, spring (March–April) drought conditions, defined using the PHDI, ranged from 42.7% wettest historically (1895–2018) in 2013 to 100% wettest historically in 2017 (Table 5). An observable, positive correlation was present between annual wetness and annual inundation extent (2013–2018), but the correlation was not significant (*R* = 0.49, *p* = 0.33). Figure 6 shows examples of mapped inundation extent across: (1) forested wetlands, (2) rivers, (3) emergent tidal wetlands, and (4) lake and emergent wetlands. In the examples, the limitations of Landsat-based inundation can be observed where errors of omission increase as rivers become narrower or wetlands become smaller. Because declines in inundation were identified using a summed two-year prior inundation extent, per-year declines in inundation (7.0% to 11.2% of inundation extent, Table 5) exceeded per-year gains in inundation (2.7% to 5.3% of inundation extent). Annual declines in inundation disproportionately occurred in the Delmarva Peninsula and eastern Virginia (Figure 7), where much of the wetland area and rivers drain into the Chesapeake Bay. The spatial pattern of annual declines in inundation extent largely matched the spatial distribution of NWI wetland density (Figure 7). Although inundation extent was dynamic, the percent of the study area mapped as inundated remained extremely stable across the six years mapped, ranging from 11.07% to 11.69%.

**Figure 8.**
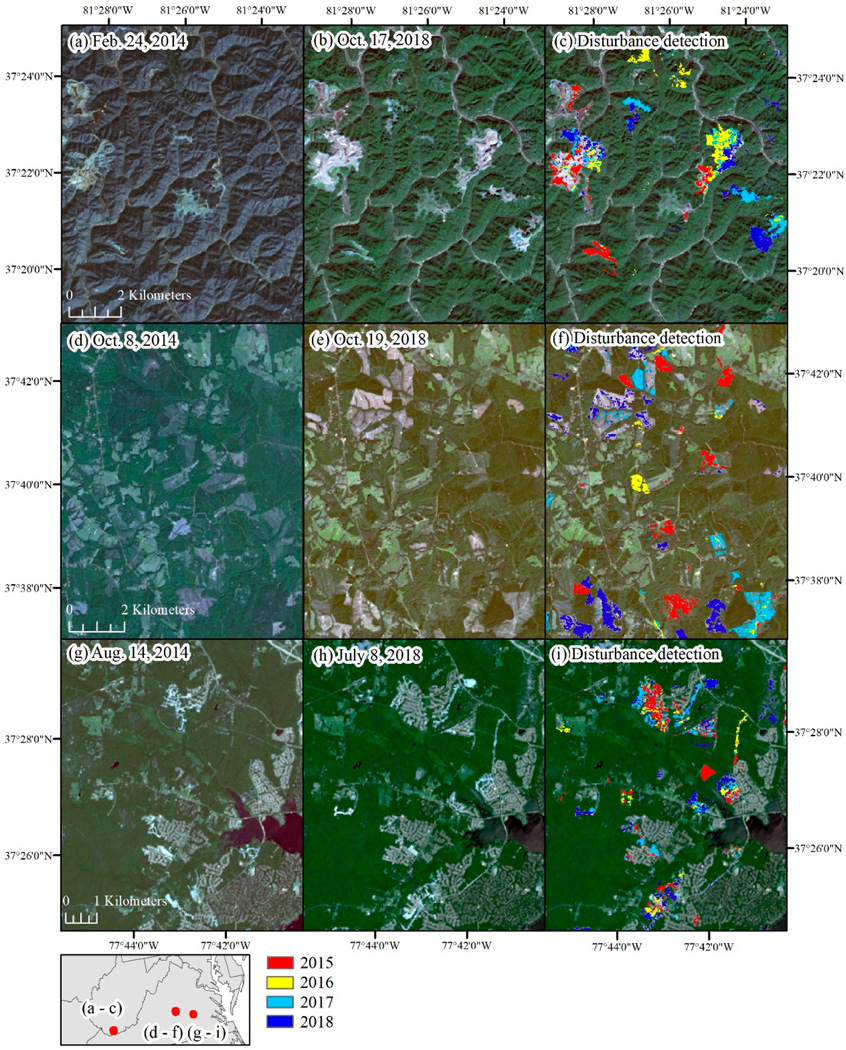
Examples of the types of disturbance detected (2015–2018) including (**a**–**c**) expansion of mining activities in West Virginia, (**d**–**f**) silviculture harvests in central Virginia, and (**g**–**i**) residential development outside of Richmond, VA.

**Figure 9.**
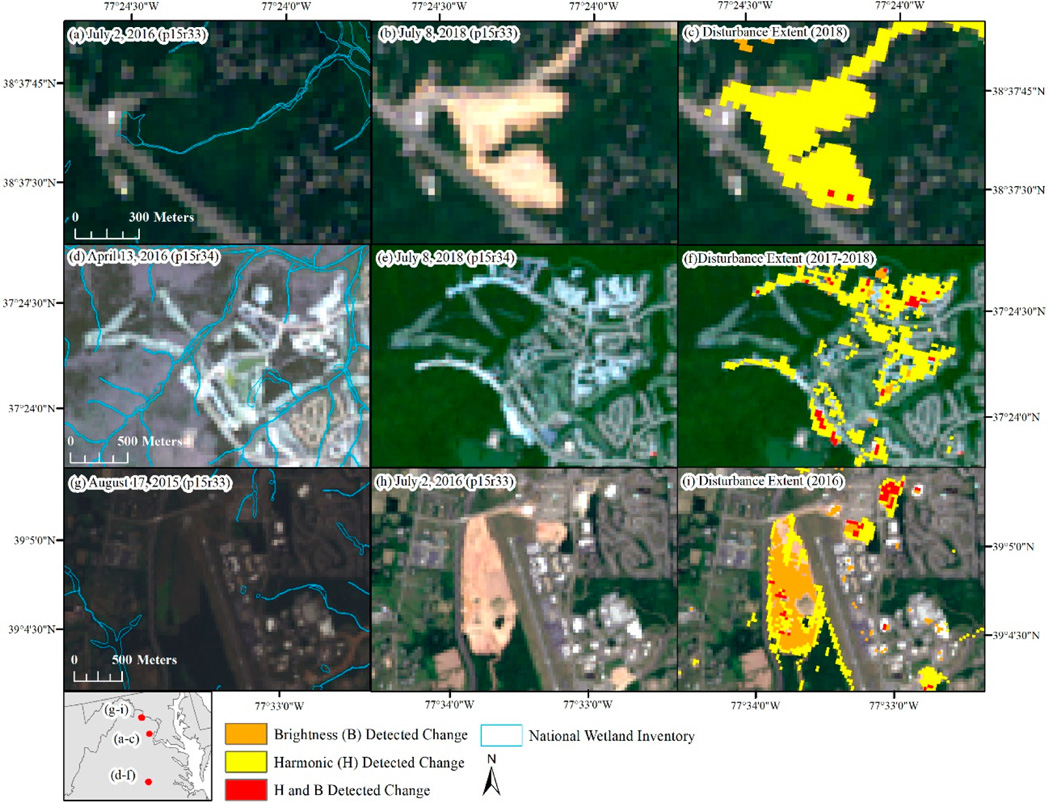
Examples of disturbance extent as mapped by change in brightness, harmonic change detection and both approaches showing the pre-disturbance imagery (**a**,**d**,**g**), post-disturbance imagery (**b**,**e**,**h**), and mapped disturbance extent (**c**,**f**,**i**). p: path, r: row.

The percent of the study area identified as disturbed ranged from 0.24% in 2016 to 0.35% in 2015 and 2018. Much of the disturbance across the region was focused in Virginia, east of the Appalachian Mountains (Figure 7). The type of disturbance was typically identifiable from the raw Landsat imagery and tended to be locally specific, with mining expansion dominating disturbance in West Virginia, silviculture disturbance dominating south-central Virginia, and residential and commercial development common around Richmond, VA, west of Dulles Airport (Ashburn, Leesburg, VA), and Baltimore, MD (Figure 8). We examined the distribution of disturbances by pre-disturbance land cover type and found 59% occurred in areas that were previously forested, 14% occurred in areas that were classified as low- to high-intensity development, and 15% occurred in areas that were classified as either hay/pasture or cultivated crops (Table 6). Examples of specific disturbance events are shown in Figure 9. In the examples, the brightness approach helped identify areas that were low in vegetation prior to experiencing further disturbance (Figure 9).

**Figure 10.**
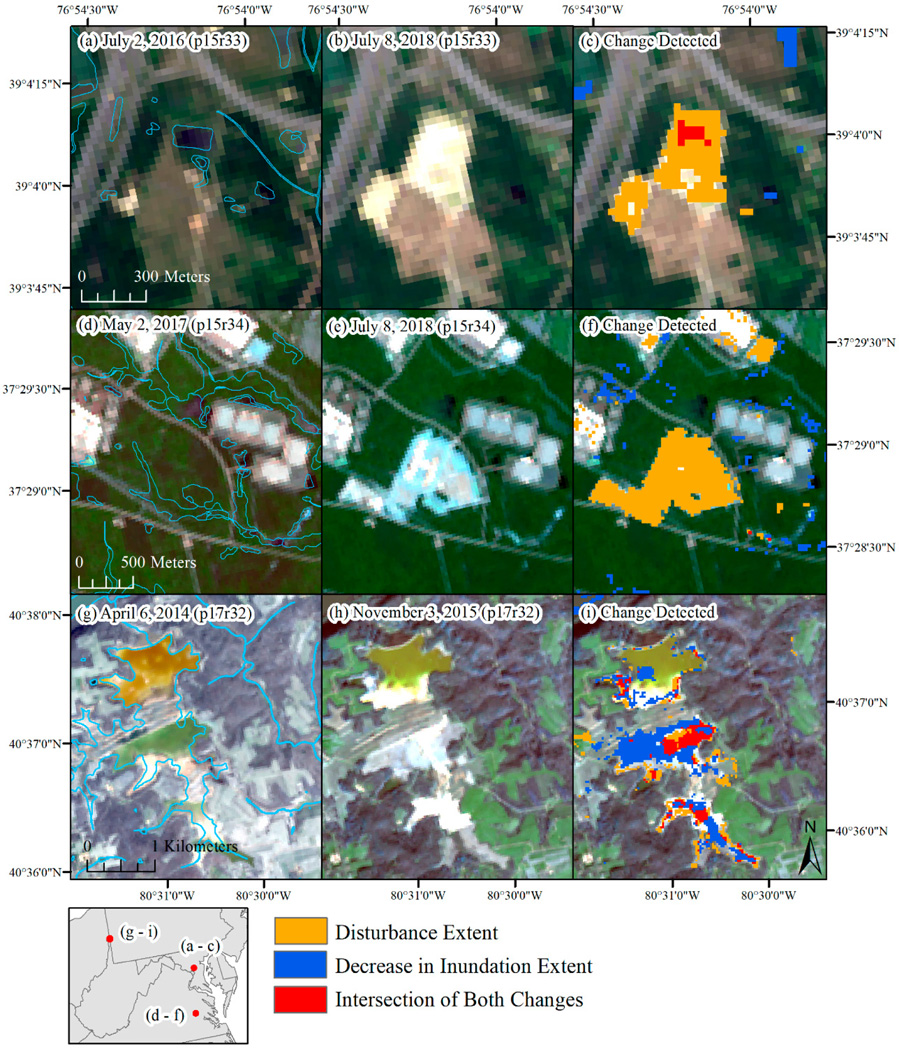
Examples of how the decrease in inundation extent and disturbance extent co-occur when impacts to water resources occur. Examples are included from Maryland (**a**–**c**), eastern Virginia (**d**–**f**), and western Pennsylvania (**g**–**i**). Light blue lines show National Wetland Inventory dataset boundaries. p: path, r: row.

The intersection of inundation loss and disturbance represented 1% or less of the annual inundation loss (0.58% to 1.12%, Table 5), meaning that ~99% of the decline in inundation extent occurred without a co-occurring disturbance event. Across the region and between 2015 and 2018 a total of 108.6 km^2^ showed a co-occurrence of inundation loss and disturbance, indicating potential anthropogenic impacts to aquatic resources (Table 5). Examples where disturbance occurred, including disturbance to water features, are shown in Figure 10. The examples show how the relative importance of disturbance, inundation loss, and the co-occurrence of both, played a variable role depending, in part, on the size of the water feature (Figure 10). To better characterize these potential impacts, the intersection of the NWI dataset and the disturbance extent was also evaluated, which totaled 186 km^2^. Core areas of disturbance were identified by internally buffering the disturbance impact by 30 m to account for mixed pixels, after which the potential impact to aquatic resources decreased to 33.4 km^2^ (Table 5), suggesting that disturbance near aquatic resources likely contributes to much of the potential impact. The intersection of disturbance with inundation loss indicated that potential impacts to water resources were more common in southeastern Virginia and the Delmarva Peninsula (Figure 7). The spatial pattern is very similar using the NWI dataset or the internally buffered disturbance extent. By wetland type, the potentially impacted wetlands were dominated by freshwater forested/shrub wetland, followed by riverine wetlands and freshwater ponds (Table 7). Many of the forested/shrub wetland and riverine wetlands are buffered streams included in NWI V2 [56] but not in NWI V1.

Finally, the potential role of watershed position was considered. Headwaters represented 56% of the study area. A minority of inundation extent (8.3%), as mapped by Landsat, occurred in headwaters, but a larger percent (46.5%) of the annual decline in inundation extent was mapped as occurring in the headwaters. A slightly disproportionately high amount of the disturbance (60.4%) extent was documented in the headwaters, while 54.8% of the co-occurrence between inundation decline and disturbance was mapped in the headwaters (55.2% of the co-occurrence between the NWI dataset and disturbance was mapped in the headwaters).

## 4. Discussion

Past studies that track the extent and cause of wetland change (from one wetland type to another) and wetland loss (conversion to upland) across the eastern United States are limited but suggest that wetland losses have outpaced wetland gains [9], and that the impact of anthropogenic activities on wetlands remains poorly understood [77]. In this study, by concurrently tracking all types of disturbance and changes in inundation extent, 95% of permitted wetland losses were identified. The distribution of disturbances was very uneven across the Mid-Atlantic region with central and eastern Virginia experiencing a disproportionate amount of the disturbance. Residential and urban development, common in eastern Virginia, was also common in the suburbs of Washington, D.C., and Baltimore. Over the four years examined, the percent of the Mid-Atlantic region mapped as disturbed, not limited to disturbance in wetlands, averaged 0.32% of the study area per year. This estimate is consistent with national estimates of land cover change. The National Land Cover Dataset (NLCD) documented a 0.25% change per year across the conterminous United States between 2000 and 2011 [55], and another study estimated an average of 0.34% change per year from 1973 to 2000 across the conterminous United States [78]. The major sources of disturbance identified in the analysis, including residential and commercial development, silviculture, and mining, were consistent with the findings of others [24,79,80]. Like the pattern of disturbance, changes in inundation extent were also uneven across the study area with much of the variability in inundation focused in eastern Virginia and the Delmarva Peninsula. This spatial pattern was very similar to that observed by Nielson et al. [24] who used Landsat to model wetland change between 1990 and 2000, suggesting that the spatial distribution of dynamic inundation extent and wetland loss has remained consistent over time.

Geographical overlap in the concentration of disturbance activities and areas with a higher density of wetlands and dynamic inundation extent means that wetland protection and attention to sustainable development may be particularly important in these regions. This conclusion is further substantiated by the findings of the U.S. Fish & Wildlife Service Status and Trends reports that have documented changes to wetlands concentrated along the Atlantic Ocean and Gulf of Mexico [80]. These findings also have potential implications for restoration activities. An improved understanding of the spatial distribution of wetland loss and the types of wetlands that are being lost can help guide restoration priorities [20] to maximize retention of the ecosystem services provided by these wetlands [3,4].

As cloud-based platforms, like the Google Earth Engine, become more advanced and accessible, large-scale, automated efforts that process thousands of images are likely to become the new “normal” in remote sensing of change. One of the challenges of applying algorithms across diverse regions is that the compilation of adequate training datasets can be time-consuming, particularly for temporary conditions, such as disturbance, or non-permanent waterbodies. Requiring training data can also make it challenging to scale or transfer an approach to a new area. In addition, computing may also be a challenge. For instance, Google Earth Engine currently limits the size of objects to be cached, meaning that the number of training points may be limited by memory, making the platform less suited for memory-intensive, machine-learning algorithms [81]. In generating disturbance and inundation extent in this analysis, the approaches were largely automated, meaning that the DSWE, the harmonic change detection algorithm, and an increase in pixel brightness were all run without training data. This approach allowed us to process thousands of Landsat images relatively easily. However, the unsupervised approaches alone were not adequate to map the object of interest while minimizing erroneous change detection. Instead, supervised classification outside of Google Earth Engine was needed to improve outputs, for example when applying DSWE to Landsat OLI and defining the spectral window of disturbed pixels.

Identifying and limiting error and other sources of uncertainty was a major component of this analysis. Some of the error encountered was at an image scale. For example, a limited number of images commonly classified much of an entire image as change using the harmonic change detection algorithm, or as low-moderate potential wetlands using the DSWE algorithm. We attribute this source of uncertainty to residual error within the Landsat image collections. Higher rates of error in a select number of images can potentially be attributed to a higher root mean square error (RMSE), errors in converting the raw images to surface reflectance, poor or uneven atmospheric conditions, or residual cloud or cloud shadow occurring after the application of cFMask [59,82]. Many of these errors are likely observable when working with individual images but may not be distinguished when working with many images. The influence of these erroneous images was reduced by requiring multiple disturbance flags and multiple observations of inundation per year. However, error was also related to the observation count. This meant that erroneously detected change tended to be higher in the overlapping portions of the Landsat path/rows (Figure 1), but also that cloud cover increased omission error for inundation extent, particularly for wetlands that might be inundated only for a few weeks per year. Cloud cover, consequently, likely weakened the relationship between inundation extent and climate indices, in that the wetter the year was, the more cloud cover, and consequently the less clear the images may have been during peak hydrological conditions. Image-based and cloud-cover related sources of error are currently intrinsic to the Landsat surface reflectance image collections, and therefore will continue to be a challenge for most Landsat-based change detection algorithms.

In addition to encountering error at an image scale or related to observation timing and count, multiple sources of spectral confusion were also encountered that introduced error in classifying disturbance or inundation extent. For instance, to map inundation extent required a substantial effort to eliminate error related to topographic shading and falsely mapped inundation, while retaining highly ephemeral, forested wetlands. In response to these sources of error, filtering inundation using rule sets specific to ecoregions was critical, as was applying a slope threshold mask. Another source of error encountered was spectral confusion between suburban areas and forested wetlands, which represent mixed pixels. This source of error was reduced by using the BU3 index [66], but some spectral confusion remained.

In mapping disturbance extent, agricultural fields originally have a substantial amount of error. This error occurred when a field was left fallow for a year or two or when the crop type, and corresponding timing of the peak greenness, changed. This source of error was most problematic across central Virginia and the Delmarva Peninsula. To address this source of error filters were added for peak greenness in the year following the disturbance year which greatly reduced, but did not eliminate, this source of error. Because development of formerly agricultural land was quite common, this cover class could not be masked. In addition, while residential and commercial development was common within and near the edges of towns and cities across the Mid-Atlantic region, erroneously mapped disturbance was also found in high-density urban areas where disturbances were not visible in the raw Landsat imagery. This source of error may be attributable to building shadows that are likely to change based on the day of year and time of day the image was collected. Consequently, a pixel’s brightness may show more variability than expected in a densely developed area. Similarly, erroneously detected disturbance was also common in tidally influenced areas where light, sandy soils become intermittently visible. Further, a documented disturbance, even in the presence of a wetland, may not necessarily result in a conversion of that wetland to upland. In the case of silviculture harvests, for example, wetland hydrology can remain following harvest and replanting [81]. Despite these sources of error and uncertainty, adequate accuracy of both inundation and disturbance extent products was obtained, demonstrating that steps can be taken to limit the influence of errors related to both the Landsat image collection, as well as spectral confusion.

Although Landsat was too coarse in spatial resolution to monitor the inundation patterns of narrow rivers, streams, or smaller (<1 ha) wetlands, disturbance around or over these water features was typically larger in extent and can be effectively identified using the Landsat archive. However, incorporating additional sources of imagery may also help overcome some intrinsic limitations of Landsat. For instance, using a SAR sensor, such as Sentinel-1 [83,84] or, in the future, the NASA-ISRO Synthetic Aperture Radar (NISAR) [85], could help overcome challenges induced by cloud cover, and enable seasonal variability in inundation extent to be tracked, potentially identifying declines in inundation extent at a temporal resolution finer than an annual time step. Sentinel-2 can also be used to effectively map inundation extent [40]. The inclusion of Sentinel-2 could help increase the number of observations near the seasonal peak in inundation extent. Both Sentinel-1 and Sentinel-2 can also potentially increase spatial resolution, relative to Landsat, making it possible to more accurately map the edges of larger waterbodies, and detect narrower and smaller (<1 ha) waterbodies. However, without a 10 m DEM, an alternative approach to reducing commission error in mountainous areas would be needed.

## 5. Conclusions

Conserving and effectively managing wetland ecosystems at a regional to national scale will require monitoring wetland extent, as well as distinguishing natural declines in wetland extent, attributable to droughts or sea level rise, from declines in wetland extent, attributable to changes in land use. Although variability in climate conditions can make it harder to identify and differentiate anthropogenic disturbance activities [86], concurrently monitoring inundation extent and disturbance enabled us to identify potential anthropogenic impacts on water resources. Further, disturbance extent paired with a wetland dataset (e.g., NWI) enabled the loss of small (<1 ha) wetlands or narrow streams to be identified. Across the Mid-Atlantic region, the spatial distribution of disturbance and inundation change was consistent between years, with southeastern Virginia showing the greatest density of both types of change. While cloud-based platforms increasingly permit big data approaches to monitor and analyze landscape changes, using thousands of Landsat images to track disturbance and inundation required rigorous approaches to identify and minimize erroneous images and spectral confusion across the diversity of the Mid-Atlantic region. Wetlands provide a multitude of ecological, economic, and social benefits. Progress in approaches to monitor wetland extent, as well as the potential cause of changes in wetland extent, will enable stakeholders to make informed, strategic decisions in a cost-efficient manner.

## Figures and Tables

**Table 1.** The number of Landsat Thematic Mapper (TM), Enhanced TM plus (ETM+) and Operational Land Imager (OLI) images used per-year for the inundation extent and per sensor for the harmonic and brightness outputs. The harmonic algorithm used images from January 2000 through December 2019, and the brightness algorithm used images for the years of interest and three years prior. Per year outputs (2015–2018) were generated within a given algorithm run.

Algorithm	Imagery Year(s)	Output Year(s)	TM (Image Count)	ETM+ (Image Count)	OLI (Image Count)	Total (Image Count)
Inundation Extent	2013	2013	~	154	85	239
Inundation Extent	2014	2014	~	201	189	390
Inundation Extent	2015	2015	~	175	205	380
Inundation Extent	2016	2016	~	184	203	387
Inundation Extent	2017	2017	~	157	221	378
Inundation Extent	2018	2018	~	165	183	348
Inundation Extent	2013–2018	2013–2018	~	1036	1086	2122
Brightness	2012–2019	2015–2018	~	3673	3540	7213
Harmonic	2000–2019	2015–2018	5037	9394	3525	17,956

**Table 2.** The per-pixel tests applied to determine if a Landsat pixel contained water. Band and index values were multiplied by 10,000 for data efficiency purposes.

Test	Landsat ETM+	Landsat OLI
Test 1	mNDWI > 123	mNDWI > 123
Test 2	MBSRV > 0	MBSRV > 0
Test 3	AWESH > 0	AWESH > 0
Test 4	mNDWI > −400, SWIR1 < 900, NIR < 1500, NDVI < 6000	mNDWI > −4400, SWIR1 < 900, NIR < 1500, NDVI < 6500
Test 5	mNDWI > −5000, SWIR1 < 3000, SWIR2 < 1000, NIR < 2500, NDVI < 4000, B < 1000	mNDWI > −5000, SWIR1 < 3000, SWIR2 < 1000, NIR < 2500, NDVI < 5500, B < 1000, BU3 < 1600
Test 6		G < 480, NIR < 2500, NDVI < 5500, BU3 < 1600

Enhanced Thematic Mapper plus (ETM+), Operational Land Imager (OLI), MNDWI: modified Normalized Difference Wetness Index, MBSRV: Multi-band Spectral Relationship Visible, AWESH: Automated Water Extension Shadow, SWIR: shortwave infrared, NIR: near-infrared, NDVI: Normalized Difference Vegetation Index, B: blue, G: green, BU3: 3 Band Index.

**Table 3.** Accuracy of inundation extent mapped with Landsat Enhanced Thematic Mapper plus (ETM+), Landsat Operational Land Imager (OLI), and Landsat ETM+ and OLI combined.

	Landsat ETM			Landsat OLI			ETM-OLI Combined		

	Reference - Water	Reference - Upland	Total	Reference - Water	Reference - Upland	Total	Reference - Water	Reference - Upland	Total
**Landsat - Water**	6096	58	6154	6027	274	6301	6793	294	7087
**Landsat - Upland**	1292	7641	8933	1383	7456	8839	979	7626	8605
**Total**	7388	7699	15,087	7410	7730	15,140	7772	7920	15,692

**Omission Error (%)**			17.5			18.7			**12.6**
**Commission Error (%)**			0.9			4.3			**4.1**
**Overall Accuracy (%)**			91.1			89.1			**91.9**
**Dice Coefficient (%)**			90.0			87.9			**91.4**

**Table 4.** The accuracy of the annual disturbance outputs using the harmonic approach, brightness approach and the combined (B-H) approach.

	Harmonic Approach			Brightness Approach			B-H Approach		

	Reference - D	Reference - UD	Total	Reference - D	Reference - UD	Total	Reference - D	Reference - UD	Total
**Landsat - D**	1978	49	2027	1191	9	1200	2290	44	2334
**Landsat - UD**	733	3553	4286	1520	3593	5113	421	3558	3979
**Total**	2711	3602	6313	2711	3602	6313	2711	3602	6313

**Omission Error (%)**			27.0			56.1			**15.5**
**Commission Error (%)**			2.4			0.8			**1.9**
**Overall Accuracy (%)**			87.6			75.8			**92.6**
**Dice Coefficient (%)**			83.5			60.9			**90.8**

D: disturbed, UD: undisturbed.

**Table 5.** Summary of annual products. Historical (1895–2018) Palmer Hydrological Drought Index (PHDI, March–April) percent quantifies how the values compare to historical conditions. Total area with both disturbance (Disturb.) and inundation (Inun.) loss or the National Wetland Inventory (NWI) present are shown in km^2^ as well as a percent of the decline in inundation.

Year	PHDI (March-April)	Historical PHDI (%)	Inun. Extent (km^2^)	Inundation Loss (% of Water) (% of SA)	Disturb. Extent (km^2^) (% of SA)	Inun. Loss and Disturb. (% of Water Loss)	Inun. Loss - Disturb. Intersect (km^2^)	NWI - Disturb. Intersect (km^2^)	NWI - Disturb. (−30 m Buffer) Intersect (km^2^)
2013	0.24	42.7	36,881						
2014	1.22	57.3	38,001						
2015	1.18	56.5	38,287	7.0 (0.80)	1221.4 (0.35)	1.12	27.6	50.1	8.2
2016	3.29	92.7	38,947	8.4 (0.99)	825.1 (0.24)	0.87	25.9	34.7	6.6
2017	4.15	100	38,084	10.6 (1.21)	1117.3 (0.32)	0.85	31.9	45.4	10.8
2018	2.69	86.3	37,284	11.2 (1.25)	1216.3 (0.35)	0.58	23.3	55.5	7.8
2015–2018							**108.6**	**185.7**	**33.4**

Inun: inundation, SA: study area, PHDI: Palmer Hydrological Drought Index (March–April).

**Table 6.** The distribution of disturbance by pre-disturbance land cover type, defined by the National Land Cover Dataset (NLCD) 2013, and the percent of each cover type across the study area mapped as disturbed during the four-year time period (2015–2018).

NLCD (2013)	Disturbance Distribution (%)	Proportion of Cover Type Disturbed (%)
Forest	59.0	1.3
Developed (low to high intensity)	14.1	1.6
Hay/Pasture	8.1	0.8
Cultivated Crops	6.8	1.2
Shrub	4.1	2.6
Woody Wetlands	3.4	1.4
Grassland/Herbaceous	1.7	1.4
Barren	1.5	4.2
Open Water	0.7	0.1
Emergent Wetlands	0.6	0.9

**Table 7.** The distribution of the National Wetland Inventory (NWI) dataset and Landsat disturbance (2015–2018) intersection by NWI wetland type.

Wetland Type	NWI - Disturbance Intersect (km^2^)	NWI - Disturbance (−30 m buffer) Intersect (km^2^)
Freshwater Forested/Shrub Wetland	115.6	27.4
Riverine	22.0	1.2
Freshwater Pond	11.1	0.5
Estuarine and Marine Wetland	10.2	1.4
Lake	9.8	2.6
Freshwater Emergent Wetland	9.7	0.4
Estuarine and Marine Deepwater	6.5	0.3
Other	0.7	0.03

## Appendix A

**Table A1.** Landsat OLI images from which the training points were extracted. The points were used only to help refine the harmonic and brightness algorithm outputs.

Landsat Path/Row (p/r)	Image Acquisition Date	Disturbed Points (count)	Non-Disturbed Soil Points (count)
p15r31	23-Sep-17	43	669
p15r31	21-May-18	53	670
p15r31	8-Jul-18	71	668
p15r33	18-Mar-18	100	600
p15r33	8-Jul-18	100	599
p15r33	12-Oct-18	100	600
p16r34	10-Jun-17	48	706
p16r34	26-Apr-18	55	707
p16r34	4-Nov-18	47	707
p17r32	26-Mar-16	73	726
p17r32	20-Aug-17	80	730
p17r32	11-Nov-18	77	730
p18r34	8-Apr-18	66	503
p18r34	29-Jul-18	82	514
p18r34	17-Oct-18	119	498
	**Total**	**1114**	**9627**

p: path, r: row.

**Table A2.** High resolution images (HR) used in the inundation extent validation and the corresponding Landsat Enhanced Thematic Mapper plus (ETM+) and Landsat Operational Land Imager (OLI) images. Image value refers to the high-resolution file name.

HR Date	Sensor	Date (ETM+)	Path/Row (ETM+)	Date Gap (ETM+)	Date (OLI)	Path/Row (OLI)	Date Gap (OLI)	State	Image
26-Jan-18	WV3	14-Jan-18	p14r33	−12	21-Dec-17	p14r33	−36	DE	P1
6-Apr-15	WV3	12-Apr-15	p14r33	6	4-Apr-15	p14r33	−2	DE	P1
6-Apr-15	WV3	12-Apr-15	p14r33	6	4-Apr-15	p14r33	−2	DE	P2
20-Feb-17	WV2	28-Feb-17	p14r33	8	20-Feb-17	p14r33	0	MD	P6
19-Feb-19	WV2	25-Feb-19	p15r33	6	10-Feb-19	p14r33	−9	MD	P1
19-Feb-19	WV2	25-Feb-19	p15r33	6	10-Feb-19	p14r33	−9	MD	P2
20-0ct-17	WV3	17-Oct-17	p15r33	−3	25-Oct-17	p15r33	5	MD	P1
20-Jan-18	WV3	14-Jan-18	p14r33	−6	28-Dec-17	p15r33	−23	MD	P3
20-Jan-18	WV3	14-Jan-18	p14r33	−6	28-Dec-17	p15r33	−23	MD	P7
23-Nov-18	WV2	21-Nov-18	p15r32	−2	22-Nov-18	p14r32	−1	PA	P1
23-Nov-18	WV2	14-Nov-18	p14r32	−9	22-Nov-18	p14r32	−1	PA	P4
29-Nov-17	WV2	4-Dec-17	p15r31	5	25-Oct-17	p15r31	−35	PA	P1
18-Oct-18	WV3	18-Oct-18	p17r32	0	10-Oct-18	p17r32	−8	PA	P3
18-Oct-18	WV3	18-Oct-18	p17r32	0	10-Oct-18	p17r32	−8	PA	P7
18-Oct-18	WV3	18-Oct-18	p17r32	0	10-Oct-18	p17r32	−8	PA	P10
18-Oct-18	WV3	18-Oct-18	p17r32	0	10-Oct-18	p17r32	−8	PA	P13
17-Aug-17	WV3	23-Aug-17	p14r32	6	22-Aug-17	p15r32	5	PA	P1
23-Aug-18	WV2	16-Jul-18	p15r33	−38	9-Aug-18	p15r33	−14	VA	P2
23-Aug-18	WV2	9-Sep-18	p15r33	17	25-Aug-18	p15r33	2	VA	P4
23-Aug-18	WV2	16-Jul-18	p15r33	−38	25-Aug-18	p15r33	2	VA	P8
21-Nov-18	WV2	28-Nov-18	p16r34	7	4-Nov-18	p16r34	−17	VA	P1
21-Nov-18	WV2	28-Nov-18	p16r35	7	4-Nov-18	p16r35	−17	VA	P4
9-Mar-17	WV3	16-Mar-17	p14r34	7	20-Feb-17	p14r34	−17	VA	P2
9-Mar-17	WV3	16-Mar-17	p14r35	7	20-Feb-17	p14r35	−17	VA	P4
9-Mar-17	WV3	16-Mar-17	p14r35	7	20-Feb-17	p14r35	−17	VA	P6
4-Oct-18	WV3	29-Oct-18	p14r34	25	21-Oct-18	p14r34	17	VA	P2
1-Dec-17	WV3	27-Nov-17	p14r34	−4	26-Nov-17	p15r34	−5	VA	P5
1-Dec-17	WV3	27-Nov-17	p14r34	−4	26-Nov-17	p15r34	−5	VA	P7
20-0ct-17	WV3	31-Oct-17	p17r34	11	7-Oct-17	p17r34	−13	WV	P3
20-0ct-17	WV3	31-Oct-17	p17r34	11	7-Oct-17	p17r34	−13	WV	P7
20-0ct-17	WV3	31-Oct-17	p17r34	11	7-Oct-17	p17r34	−13	WV	P10
20-Nov-17	WV3	31-Oct-17	p17r33	−20	24-Nov-17	p17r33	4	WV	P1

WV3: WorldView-3, WV2: WorldView-2, p: path, r: row.

**Table A3.** Distribution of newly disturbed validation points and the Landsat Operational Land Imager (OLI) images used to identify the change. A total of 150 no change points were randomly selected for each year (2015–2018) and each path/row (n = 3600).

Path/Row	Image Date	Disturbed Soil Points	Path/Row	Image Date	Disturbed Soil Points
p15r31	24-Apr-14		p16r34	10-Jun-17	162
p15r31	29-May-15		p16r34	19-Oct-18	59
p15r31	13-Apr-16		p17r32	6-Apr-14	
p15r31	23-Sep-17	41	p17r32	3-Nov-15	131
p15r31	8-Jul-18		p17r32	5-Nov-16	171
p15r33	24-Apr-14		p17r32	24-Nov-17	80
p15r33	14-Aug-14		p17r32	11-Nov-18	63
p15r33	17-Aug-15	150	p18r34	24-Feb-14	
p15r33	2-Jul-16	247	p18r34	20-Sep-14	
p15r33	22-Aug-17	172	p18r34	15-Mar-15	
p15r33	8-Jul-18	167	p18r34	23-Sep-15	70
p15r34	14-Aug-14		p18r34	18-Apr-16	169
p15r34	17-Aug-15	156	p18r34	30-Oct-17	130
p15r34	13-Apr-16	98	p18r34	17-Oct-18	159
p15r34	2-May-17	164	**Total**	**2015**	546
p15r34	8-Jul-18	185		**2016**	793
p16r34	8-Oct-14			**2017**	749
p16r34	20-May-15	39		**2018**	633
p16r34	7-Jul-15			**2015–2018**	2711
p16r34	29-Oct-16	98			

p: path, r: row.
